# Supporting Clinician-Scientist Development in Child Psychiatry: A Four-Domain Model for Individual or Programmatic Self-Reflection

**DOI:** 10.3389/fpsyt.2021.651722

**Published:** 2021-03-31

**Authors:** Amanda Calhoun, Olivia D. Herrington, James F. Leckman, Andrés Martin

**Affiliations:** Child Study Center, Yale School of Medicine, New Haven, CT, United States

**Keywords:** clinician scientist workforce, research training and mentoring, child and adolescent psychiatry, qualitative methods, scientific independence

## Abstract

**Background:** The Albert J. Solnit Integrated Training Program (AJSP) is a novel educational initiative designed to prepare physician-scientists for independent careers in the investigation and treatment of childhood psychiatric disorders.

**Methods:** We conducted a qualitative study to explore the impact and active components of the AJSP through hour-long individual interviews of its enrollees and graduates. We were specifically interested in identifying individual or programmatic traits for success that could be replicated elsewhere. As components of our theoretical framework, we used sources on Strength, Weakness, Opportunity, and Threat (SWOT) Analysis as applied to healthcare, and on mentorship and career development as pertaining to child and adolescent psychiatry (CAP).

**Results:** Thirty-four individuals matriculated into the AJSP between 2004 and 2020, 33 (97%) of whom participated. Through iterative thematic analysis, we developed a model consisting of quadrants resulting from the intersection of a developmental perspective (spanning professional or personal spheres) and a reflective direction (with outward- or inward-facing vantage points). The model can be of practical utility through putative questions that trainees/program leaders could ask themselves by using the four domains as points of departure: (I) Individual: “Is becoming a clinician-scientist right for me?”/“What traits are we looking for in prospective applicants?”; (II) Program: “Is this the right program for me?”/“What is the right balance between structure and freedom for trainees to thrive in?”; (III) Mentorship: “What is the right number and constellation of mentors for me?”/“How can we optimize our experience and backgrounds toward the benefit of our trainees?”; and (IV) Charting Course: “Who do I want to become?”/“How can we help our charges embrace, find, or reconnect with their true vocation?”

**Conclusion:** Our analytic approach can help identify, refine, and replicate programs that are urgently needed to increase the workforce of clinician-scientists dedicated to improving the well-being and mental health of children and families. The model we describe can be fruitfully applied to the self-reflection by individuals or program leaders. Although based on a single program with very specific goals, the model could also be applied to other training initiatives within psychiatry—and beyond.

What you get by achieving your goals is not as important as who you become by achieving your goals.

– Henry David Thoreau (1)

## Introduction

More physician-scientists are needed to pursue research careers in understanding and addressing the pathogenesis, treatment, and prevention of child and adolescent mental health conditions (2). The shortage of clinician-scientists dedicated to the mental health needs of children is urgent given the “heavy burden on young minds” at a global level (3, 4). There is a need for combined research and clinical educational programs that can introduce scientific advances and prepare physician-scientists for interdisciplinary careers through the acquisition of advanced research skills and working collaborations with clinicians and scientists in related fields. The traditional model of training in child and adolescent psychiatry (CAP) provides opportunities for medical students and residents to participate in child and adolescent clinical services but does not typically encourage CAP trainees to pursue formal research training. This unfortunate reality has had a significant impact on the minting of new doctors in the “endangered but essential” (5) physician-scientist mold.

Innovative approaches to increase exposure to CAP during medical school have proven effective in raising awareness about the field, including its research opportunities (6), and in enhancing recruitment into psychiatry (7). In 2004, we launched a model curriculum aimed at providing newly graduated physicians with an integrated program combining training in CAP with early and ongoing formal and hands-on training in research. This program, crafted with the assistance of a national task force appointed by the American Academy of Child and Adolescent Psychiatry (AACAP), has been continuously funded since its inception through grants from the National Institute of Mental Health.

In an earlier study based on data from the program's first 15 cohorts (2), we documented that graduating medical students enrolled in this integrated training program, when compared to peers who had been similarly ranked in original match lists but ultimately pursued residency programs elsewhere, were more likely to become clinician-scientists dedicated to careers in CAP and had higher metrics of academic productivity and scientific independence. Specifically, integrated program participants outperformed their peers across the outcomes of: (1) AACAP development career awards, with a higher number of awards per recipient and a shorter time to first award; (2) PubMed entries, with more publications, more first-authored publications, a higher h-index, and a shorter time to first publication; and (3) higher federal grant (NIH K- or R-series) funding success rates (*p* < 0.05 for all comparisons). From a return-on-investment perspective, the 2.8 million dollars in R25 funding since 2004 has already yielded 4.6 million dollars in new grant funding. Quite aside from hard metrics and fiscal bottom lines, the program has led to the formation of a unique group of clinician-scientists with remarkable scientific creativity, innovation, and commitment.

As a complement to that report, we report here on a qualitative research study through which we sought to gain a better understanding of the program's unique characteristics—as viewed from the perspective of its most integral stakeholders. We were specifically interested in identifying individual or programmatic traits for success that could be replicated elsewhere. By including currently enrolled trainees and graduates to date, our findings incorporate the views of all but one of the entire 17-year cohort of AJSP participants.

## Methods

### Program Description and Participants

The Albert J. Solnit Integrated Training Program (AJSP) was implemented at the Yale Child Study Center in 2004. Named after the Center's third director, the AJSP integrates training in pediatrics, psychiatry, CAP, and research competencies into a 6-year continuous experience. Participants are recruited into the program after graduation from medical school and, upon completion, become board-eligible in both general and CAP. The program's overall structure and specific details are available in prior publications from our group (2, 8) and on the AJSP website (9).

Funding for the AJSP derives from a range of sources, including traditional graduate medical education training slots through Yale-New Haven Hospital and the Veteran's Administration Hospital, which, combined, provide most of the support for four of the 6 years of training. The remaining 2 years, largely devoted to protected research activities, are funded through a combination of federal and private sources: (1) an R25 grant from NIMH (MH077823), now in its 15th year, designed to target the specific research goals of the AJSP; (2) a T32 training grant from NIMH (MH018268), now in its 36th year, which supports training infrastructure across a range of relevant disciplines; and (3) a significant amount of philanthropic support.

Annually, we receive more than 400 applicants for two available training slots. We are committed as a programmatic goal to improve diversity in the CAP physician-scientist workforce. Toward that end, we actively seek applicants from ethnic or racial groups that are underrepresented in the field of medicine, including African American, Hispanic/Latinx, and Native American or Pacific Islander. Interviews are only offered to those graduating students who have displayed during their medical school training a clear interest in both child psychiatry and in research, preferably exemplified through published work. Each year, some 30 applicants are offered in-person interviews, with a formal selection process to finalize a ranked match list. Since the program's inception, we have matched the two available positions each year. To date, we have recruited 17 cohorts (*n* = 34, 2004–2026).

### Data Collection, Qualitative Analysis, and Theoretical Framework

We conducted hour-long individual interviews with each of the participants. Interviews were recorded and transcribed verbatim. Deidentified transcripts were then uploaded for analysis supported by NVivo 12 software (QSR International, Melbourne, Australia).

We analyzed the transcripts using thematic analysis (10, 11), which provides theoretical freedom and flexibility to identify commonalities, and in which writing and analyzing data occur recursively alongside one another. Our analyses were framed within a constructivist framework, which welcomes and encourages attention to *reflexivity* (12), or the investigators' personal and subjective views. Two authors (AC, OH) worked independently to identify and compare codes before removing redundancies, sharing them with the other investigators for further refinement, and finalizing them into a joint codebook of overarching domains and themes until reaching *theoretical sufficiency* (13), the point at which additional data do not contribute further to the development of a given domain or theme, or to the creation of a new one. Each key theme was supported by multiple quotations. We followed best practice guidelines for the analysis, drafting, and submission of qualitative studies (14, 15). In keeping with the tenets of participatory research (16), we value the perspective of all involved participants and invited them to review and comment on our final codes, overarching conclusions, and manuscript draft.

As components of our theoretical framework (17), we used works on Strength, Weakness, Opportunity, and Threat (SWOT) Analysis as applied to healthcare (18), and on mentorship (19) and career development (20) as pertaining to CAP.

### Ethics Approval

We obtained institutional review board approval from the Yale Human Investigations Committee (Protocol # 2000027895). Subjects were encouraged to participate but informed that their participation was not mandatory. They were aware that interviews were conducted as part of a research project and provided recorded verbal consent to participate in the study.

## Results

Thirty-four individuals matriculated into the AJSP in the 17 years between 2004 and 2020, 33 (97%) of whom agreed to participate in this study and provided consent. Participants comprised 21 graduates and 12 enrolled trainees. Sex distribution was balanced among the 33 subjects, nine of whom (27%) are members of under-represented demographic groups (five African American, three Latinx, one Native American). Two-thirds of participants have an advanced degree separate from their MD (17 PhD, 5 MPH or equivalent). Of those degrees, 17 had been conferred prior to matriculation, and 5 were earned as part of academic work during the course of the AJSP. Half of eligible participants had received financial support in the form of loan relief through the NIH Pediatric Research Loan Repayment Program.

### Four-Domain Model

Through iterative thematic analysis, we developed the model depicted in Table 1. The model comprises four domains relevant to the training of clinician-scientists in CAP: (I) Individual, (II) Program, (III) Mentorship, and (IV) Charting course. Each of the domains is a quadrant resulting from the intersection of a developmental perspective (professional or personal) and a reflective direction (outward- or inward-facing).

**Table 1:** Developmental perspectives, reflective directions, four domains, and underlying themes for clinician-scientists.

		**Reflective direction**	
		***Outward***	***Inward***
**Developmental perspective**	***Professional***	Program *Strengths* *Weaknesses* *Opportunities* *Threats* *Balance*	Charting course *Focus* *Method* *Calling* *Creation*
	***Personal***	Mentorship *Launch* *Orbit* *Landing* *Woes*	Individual *Strengths* *Weaknesses* *Opportunities* *Threats* *Balance*

#### I. Individual

The first two domains are organized using a similar “SWOT-B” rubric: (1) Strengths, (2) Weaknesses, (3) Opportunities, (4) Threats, and (5) Balance. This analysis, as applied to individuals, is outlined in Table 2 and described below.

**Table 2:** Individual characteristics: SWOT-B analysis.

**Theme**	**Construct definition**	**Representative quote(s)/*(Subtheme)***
Strengths	Internal positives and advantages; inherent to the individual	There is that evocative quote from the Indigo Girls: “There's more than one answer to these questions/Pointing me in a crooked line/And the less I seek my source for some definitive/Closer I am to fine.” We're looking for concrete answers, but the more we're committed to that concrete part, the less successful we'll be, and probably the less happy. So part of the searching theme is finding comfort and understanding and solace in the non-answers, in something not definitive. *(Comfort with uncertainty)*
Weaknesses	Internal hindrances or vulnerabilities; inherent to the individual	The research I saw frustrated me in that it was very important in expanding the frontier of our knowledge but wasn't helping patients today with how they were doing. We needed those researchers, but that wasn't me.
Opportunities	External positives; resources available for use or incorporation	Especially as people of color and as women, we should take advantage of that when we're getting ready to leave for our first faculty appointments. Because we have been given a really wonderful gift through the program; we're capable of doing really great things and people recognize that. And so, we shouldn't sell ourselves short when we're on the launching pad to start our careers.
Threats	External hindrances or realities; impinging from outside sources	As I contemplated the financial and time horizon realities, I saw that my interest in all this research stuff could well fizzle out, that I would likely become ‘just’ a clinician.
Balance	The ability to incorporate competing demands toward a point of personal equipoise	In the traditional way, you are chasing these grants, and I don't think it has to be like that. You can have a combination of different things that you like to do. You can have different kinds of balance in your clinical practice, or clinical experiences in research. You don't necessarily have to commit to a single line of research; there are different ways to participate in research, to have it remain worthwhile and fun, to be meaningful.

##### Strengths

*Patience | Comfort With Uncertainty | Grit | Tenacity With Long-Term Goals*. Participants identified several character traits that had served them well over the course of their training. *Patience* was paramount, and not only because science and scientific development take a long time, or because time was inherently necessary “to mature and tie things together,” but also because the program they had joined was similarly evolving and resolving its own developmental challenges, particularly during its early years. Progressing in lockstep with their evolving program certainly required *comfort with uncertainty*, but that trait was necessary well beyond the particularities of any given program: “There are no assurances of success, or of a given experiment even working out. You just buckle up, hold on tight, trust the process, and go go go.” Uncertainty could be not just tolerated but embraced, so long as there was a perspective that “the scientific journey may be more than its destination” and that struggle and failure are inherent to its process:

These years have been lovely, and I've found wisdom and I've been on a search, but I've also seen failure and encountered struggle, which I don't see as antithetical to wisdom or to searching. It's obviously part of what makes the search worthwhile, and the wisdom that much more profound.

*Grit*, stick-to-it-ness, and work ethic were deemed essential traits to overcome the vagaries of uncertain outcomes, as was *tenacity with long term goals* and with the inevitable (and frequent) handling of rejection in its many forms.

*Self-Directedness | Intellectual Flexibility | Psychological Adaptability*. Assertiveness, initiative, *self-directedness*, “being bold,” and “even having somewhat of a pioneer spirit or entrepreneurial style” were deemed valuable in a setting in which trainees are given considerable range in designing their own program and research goals:

Some folks need rulers and margins, they like the color-by-numbers approach. Others love the freedom of the blank page. You have to be a blank-page enthusiast to thrive around here. Or maybe to thrive as a clinician-scientist anywhere.

Having to change research and clinical settings periodically necessarily implied that *intellectual flexibility* was critically useful. As such, it behooves training environments to help trainees enhance such flexibility along the way, specifically around the dual skills of *learning to learn* and *learning with others*. The pace at which knowledge advances and the seemingly inexhaustible range of resources at our disposal require a different approach to learning. The information contained in the static books and lectures of old can no longer be today's mainstays. *Learning to learn* requires dynamic skills that are iteratively refined, lifelong, web-/distal- as well as person-based, asynchronous as well as synchronous, self-initiated, trainee-rather than trainer-centered, and responsive to actual challenges at the individual, community, and policy levels. As specialists dedicated to development and to human interaction, we are in a privileged position to learn not only *about* others but *with* others. Indeed, few of us could conceive of practicing alone: our work is multidisciplinary and relationship-based at its core.

*Psychological adaptability* was useful to acclimatize to new environments, to balance the pursuit of one's goals with the pull toward another's, to remain open to unexpected opportunities of the kind “you are never quite ready for, but where a little nudge at the right time can prove critical.” Adaptability was required not only to articulate an early scientific vision but also to change it iteratively over time. Accepting the limitations of our predictive powers required a different kind of humility: “will my endpoint 6 or 10 years from now be in alignment with my goals of today?” As program directors, we were well-positioned to support the psychological adaptation and well-being of our trainees:

My own psychotherapy and psychoanalysis, which has now been going on for a long time: that was very unexpected and ended up being a big growth driver for me. I had pigeonholed long-term therapy as something narrower, an indulgence perhaps.

*Background*. We relegate these traits to the end, as they are the less actionable ones by the time of matriculation. It would be disingenuous to deny that some participants had inherent advantages by the time they started the program, whether through prior research or clinical experience, by coming from the same institution, or by having an additional advanced degree (particularly an MD/PhD—“just to give us a ‘fighting chance’ in basic science”). However, we should note that none of these variables, including PhD status, were predictive of higher success rates in the “hard outcomes” examined in our earlier, quantitative study (2).

##### Weaknesses

The internal hindrances or vulnerabilities that we saw reflected in the interviews can be grouped into three distinct categories. The first may be construed as the reverse of the previous section, as *anti-strengths*, whether actual or perceived and self-imposed. For example, individuals who became impatient with lacking an active project or a concrete direction, those who sought shorter-term returns or early “wins,” and those frustrated with the timescale of research (particularly in the basic sciences) were much more likely to struggle:

They are clearly bright but when they came in and saw how unsatisfying science can be, how little reward there can be immediately, they just couldn't tolerate it, and abandoned it.

*Physicians' Pathophysiology*. A second category pertained less to the burdens unique to aspiring clinician-scientists, and more broadly to the toll exacted across all medical providers. Participants described a range of difficult feelings, including being overwhelmed, exhausted, unappreciated, or stretched too thin. Others reported fears of failure, confrontation, or conflict; pretending to be fine; feeling wary of a competitive environment; and having experienced (or continuing to experience) imposter syndrome. Even though several participants shared how psychotherapy had been integral to their training experience, few acknowledged personal psychopathology or its impact on their functioning. By contrast, “burnout” was a more readily used term, even as it may have conflated or overshadowed underlying struggles.

*Disambiguating Ability From Drive*. The third category may be more specifically relevant to aspiring clinician-scientists, for whom disappointments, doubts about personal ability, or a fear of letting themselves or others down may have additional downstream consequences. Even as several participants had considered quitting science at some point, few had felt comfortable articulating their doubts outside of the interview setting; it appeared easier to “blame” self-perceived limited abilities than acknowledge that they may no longer have had the drive to pursue science:

I am able to do and enjoy doing science, when guided. But do I really, truly want to be a Principal Investigator (PI) and run a lab myself? I've turned down opportunities before, admitting I couldn't do something. But what if I no longer *want* to do something? That seems much harder to accept.

##### Opportunities

Regardless of their relative strengths and weaknesses, participants shared a common sentiment of gratitude for having the opportunity to grow and develop “in our own way, without having to sacrifice our values.” Having the continuity of 6 years of training in a single program permitted trainees to explore, revisit, and refine in a recursive way “our personal narratives, so that we can work out who it is we are really aiming to become.” “Pluripotentiality” was the term used by an interviewee as a common denominator for the group as a whole, with the program's flexibility for customization “permitting us to develop however it is we want, to choose what kind of psychiatrist we want to become.” Others reflected back on their early and inchoate days, when they had questioned whether they were a good fit and had made the right decision selecting the program,

… appreciating it for taking a chance on me, because some folks just jump in and it's really clear what they're going to do from the start. But that was not me: I needed a lot of time, care, and feeding to get there.

##### Threats

Pursuit of a clinician-scientist route was described in an interview as “so much easier *not* to do.” Length of training, opportunity cost (through delayed joining of the workforce by at least 1 year), loan burden, and immigration barriers (with program eligibility restricted to U.S. citizens) were among the potentially impinging externalities that gave pause or, in some cases, led to departure from the trajectory altogether. Additional recurring concerns were the time horizon and uncertainty around scientific independence and a realistic awareness of the challenging funding environment ahead. An additional and discomforting reality was the prospect of having to compete with “pure” scientists who did not have to devote a substantial portion of their effort to clinical training and practice: “I'm up against people who dedicate 100% of their time to the lab. I simply didn't have that.”

Poor role models posed a dangerous example that could have a chilling effect. A hierarchical system was described as one that could devolve into “getting so abused in academic medicine and just keep going with it. It's incredible to me. But it is a model that we have seen in medicine over and over again.” Less egregious, but far more common, was the cautionary tale of the research life as “a grind” in which

They are chronically stressed, always working on a paper, wondering where the next grant is going to come from, feeling like they're one bad grant cycle away from not being able to pay their salary. Maybe I'm just not doing it right myself. But I haven't seen that many people who've pulled it off, to be honest.

##### Balance

The quest for an elusive point of equilibrium was at the forefront of the thoughts of most, many of whom had seen cautionary tales of clinician-scientists perceived as “productive yes, but at the price of appearing not so well adjusted.” Among the top priorities to address during training years and beyond: integrating personal and professional goals, nurturing family life, being attentive to significant others or spouses, cultivating outside interests, and, of particular pre-eminence for those with children, being the best parents they could be.

A second aspect was at the professional level, aiming to blend the clinician and scientist constituents in proportions that made best sense for each individual. The combination of the two components was a necessity going well beyond the allotment of hours or percent effort; it was about integration into a cohesive personal narrative and trajectory, as exemplified in two complementary reflections:

I'm trying to figure out how to make the training valuable in my work, because right now it's not particularly relevant. So far, I haven't yet found a way to directly make that knowledge applicable to my work.

The tension I feel is that every hour that I spend writing a paper or applying for a grant is an hour that I'm *not* spending with that family or doing the work face-to-face with these kids. That's not a fair way to think about it, because publishing does allow the work to continue through other people who are doing similar things. But I'm just not about that. I have a pressing need to help real people, right now.

#### II. Program

Table 3 outlines the “SWOT-B” analysis as applied to the program; the resulting components are described below.

**Table 3:** Program characteristics: SWOT-B analysis.

**Theme**	**Construct definition**	**Representative quote(s)**
Strengths	Internal positives and advantages; inherent to the program	I picked this program because it reminded me of the flexibility we had during medical school, where they really wanted you to pursue whatever you were interested in: “We'll give you the resources and support for you to make it happen” —that sort of “can do” vibe.
Weaknesses	Internal hindrances or vulnerabilities; inherent to the program	Spanning so many programs, we could get lost in the sauce. During intern year we didn't have many check-ins, when it was an important time to lay down the bricks for what you would be doing next. And if you needed more support, you didn't necessarily have time, energy, or courage to reach out for more support at that time because you're so involved in pediatrics and far from child psychiatry.
Opportunities	External positives; resources available for use or incorporation	Having opportunities to formally visit and spend time with, to connect with talented individuals at other institutions was one of the great and unique training opportunities of the program.
Threats	External hindrances or realities; impinging from outside sources	We had tough financial problems, which is totally understandable. Running these programs is a nightmare. Getting these things funded is a disaster. Nobody hands you a bag of money to do this stuff, so it makes sense that there were financial problems.
Balance	The ability to incorporate competing demands toward a point of programmatic equipoise	When I was getting ready to leave residency and look for jobs, coming from the program was a big stamp of approval: they recognized we are as clinically strong as we are research strong and they really hustled to try to find a position for me and make something work. Which was really, really nice.

##### Strengths

Positive comments of the program fell into four broad categories:

*Uniqueness of Structure*. With no other training program organized in quite the same way, its approach stuck out:

I've been able to customize my residency training in a way that is in line with my interests, which I don't think I would have been able to do anywhere else.

As with any idiosyncrasy, such singularity was a source of both interest and dissuasion: rather than a program of universal appeal, it became one better suited for those seeking to thrive in an environment with considerable room for customization, one that “provided the raw materials and let you ‘build your own adventure.”’ The program's unheralded amount of autonomy, particularly in its final 2 years, was described as “either thrilling or frightening, depending on your goals, but a definite line in the sand in terms of whether you want to be here.”

*Integrative Design*. Incorporating clinical and research components from the outset, the program was experienced as rigorous, broad in its training experience and settings, and allowing for the simultaneous mastery of core clinical competencies and research immersion and training:

The program allowed me to pursue research seriously. It's otherwise too easy as a physician to get swamped with clinical responsibilities and forego research.

*Developmental Perspective | Early identity formation*. There are other programs that seek to integrate clinical and research training, and participants had considered or interviewed at several of them. However, the possibilities narrowed considerably for applicants with a clear commitment to working with children and adolescents. The ability to work with children from the start was a highly valued aspect of the program, including through an internship year in pediatrics rather than medicine and through early exposures to inpatient and consultation-liaison child psychiatry. Incorporating a developmental framework that championed the unique needs of children and their families mirrored the values of an independent department of child psychiatry and resulted in a sense of hopefulness and enthusiasm for “working from the outset with the population and age groups we love, without having to wait.”

*Continuity Threads*. Given that the program spanned at least three distinct departments (pediatrics, psychiatry, and child psychiatry) and a wide range of research settings, providing continuity in training, education, supervision, and longer-term career planning became paramount for the overall experience to be summative rather than piecemeal. This was approached through longer-term outpatient clinical experiences, program leaders and mentors who were “supportive and informed, but not overbearing or micro managerial,” and by the bonding across classes that resulted from the program's small size. This cross-class peer collaboration proved essential in maintaining institutional memory and in providing for agency in shaping and refining the program:

We talked about a challenging rotation and were able, as trainees, to advocate for change. That experience was so affirming, and so different from having a more boilerplate, preordained, this-is-what-you-will-do approach to training.

##### Weaknesses

*Growing Pains*. There was a clear divide in the critiques made by respondents enrolled during the first or second half of the program's existence. Those in the early years, during which components and overall design were still in flux, expressed a deep commitment to the mission of developing not only themselves but also the program with which they were affiliating themselves: “it was about survival back then, not refinement,” “it was free-flowing, work-in-progress, only so much of which is a good thing.” The early years were about working out the kinks, both small and large:

It was a small operation back then, but none of the leaders seemed to be talking to each other so much, to the point where they did not coordinate their annual parties one year: symptomatic, but none of it malicious. It was just mind boggling how little coordination there was in those early years.

There were mistakes made early on with how funding was structured when we were on grants. Some of those administrative oversights impacted my loan repayment later on. It was solved in the end, but at the time I felt abandoned and let down by the program.

*Organization*. Although such larger, structural problems were resolved in subsequent years, the program continued to feel disjointed to some. Structural, cultural, and organizational differences across component departments could loom large; it could be easy to become disconnected, transient, floating in a no man's land or limbo, “too easy to get lost in the shuffle.” Some described feeling during their training “so special and talked up that it led to sibling rivalry with others” or, alternatively, “so overlooked that we were left off invitations and announcements.” Moreover, the program felt to some too small and insular, challenging trainees' ability to provide meaningful feedback and trusting that it would remain confidential.

*Moving From Career Prognostication to Timely Diagnosis and Treatment*. With an explicit goal to develop clinician-scientists ready for independent careers, the program understandably tries, during recruitment, to prognosticate which candidates will prove a good fit and be likely to succeed. As the training years go on, the outcomes in question become concretized in the form of awards, publications, or grants. Critical as these elements are to the success and longevity of the program, there were a number of comments about overlooking the parallel need to better “diagnose” and “treat” in a timely fashion. From a professional perspective of “career health,” going off track scientifically or contemplating dropping out (from the program or from the pursuit of science) were described as off-limit topics, too embarrassing and shameful to bring up: “but there needs to be a way not only do talk about it, but to have ‘off-ramp’ options available.” Similarly, bringing up personal mental health issues was described as “an additional burden” that could fall on trainees, rather than a routine part of the program's oversight of them.

##### Opportunities

*Scholarly Settings and Social Capital*. Respondents had made good use of a broad array of clinics, labs, and projects at their disposal. The variety and abundance was particularly relevant to less “traditional” applicants or interests, as in the case of fruitful partnerships with the humanities, or when facilitating a trajectory shift during training. Aside from the broad set of local opportunities, strategic efforts were made over the years to connect trainees with content experts or specific mentors at other institutions. Paradigmatic of this approach were visits organized each year by the program's chief residents to exemplary research settings in the region:

I really broadened my outlook, both personally and professionally, during my training years here—partly through all the opportunities that were facilitated in places and with people outside of here.

*Leveraging Resources*. From very early on, trainees were made aware of relevant opportunities for awards or grants. These included internal and external options, as well as private and public, including the federal loan repayment project. Grant-writing skills, opportunities to present in a variety of settings, an ethos that “our teachers and mentors had more confidence in us than we ever did,” and the positive reinforcement of early wins, all translated into a documented high success rate, both during training and after its completion:

Getting outside pilot funding for my own ideas during training was so important; securing those early grants helped me build confidence that I could do it and maybe take the next step.

There are so few people like us out there, that especially when people decide to stay primarily as researchers, we become like these coveted rare unicorns, it gets crazy. I think that the program really set us up to have this amazing launching pad.

##### Threats

*Funding*. The fiscal worries engendered by a costly program that relies in large measure on the soft monies of renewable federal grants and philanthropy are never entirely subdued. In the early years, those concerns were existential, with no assurance that the program could come into being (“one bad grant decision away from not”); now that the program is instantiated, the apprehension has shifted away from viability toward longevity.

##### Balance

The more readily evident sides of the equation involve its clinician and scientist components, or the harmonizing of different sites and institutional structures. But at a broader level, it may be more relevant to consider the optimal setpoint for programmatic flexibility: just how pre-determined and set (“prix fixe”) or customized (“à la carte”) should it be? Erring on the side of the former, the program could turn out to be not meaningfully different from the traditional approach that has led to such limited yields: an internship followed by 2 years of psychiatry, two of child psychiatry, and one of post-fellowship research. Alternatively, erring on the side of too much customization could lead to a disorienting experience, unstandardized training, and an ultimately unsustainable approach in which “anything goes.”

#### III. Mentorship

The third domain encompasses four themes organized along an *Ignition Sequence* metaphor: (1) Launch, (2) Orbit, (3) Landing, and (4) Woes, as outlined in Table 4 and described below.

**Table 4:** Mentorship: ignition sequence.

**Theme**	**Construct definition**	**Representative quote(s)**
Launch	Mentorship is a labor of love, its success as likely to depend on the labor as on the love parts of the equation; it requires focus and care hovering in balance over two separate domains: that of the *other's work* and that of the *other*.	They seemed more hands-on, even though what they were doing clinically and research-wise didn't totally line up with my specific interest. But there were enough parallels and this person had a large research group, much more formalized structures, lab meetings, presentations, one-on-one meetings, work with postdocs and people at other levels. And that worked well for me. The fit with the person, I think 9 times out of 10 is more important than the scientific fit.
Orbit	Mentorship provides a means through which to identify, coax, and ultimately ignite the dormant potential of another. A mentor can see what is best in a mentee and help propel it forth, often seeing this potential well before the mentee can, sometimes even as the latter's self-doubts continue.	Emotions ran high and it was stressful when I presented. But I got excellent feedback and felt like, “Oh, this is how it's supposed to be. This is how I'm actually going to refine things and move forward rather than all those muddled attempts I had made before.” It was the first click that this research thing could actually happen for me. It was so helpful having a community of folks that have gone through it and made it to the other side, and who know you well and can give you honest feedback too.
Landing	Internalization can be seen as providing a useful metric for the success of the experience: Those individuals capable of invoking and making use of the other have been effectively mentored. A natural corollary is that in the process, they themselves have become mentors to others and the cycle and its transmission of values have effectively moved forth.	Have I ended any mentor experience, any mentor relationships? I have *moved on* to different projects and mentors –but I have not formally *ended* anything.
Woes	Upon first signing up to the task, mentors should remember that no matter how talented, their charges may be quite vulnerable early on. They would do well to recall how vulnerable they themselves once were, and recognize, with more awe than fear, that their influence can be enormous, but not de facto for the better.	I really wasn't well equipped to take advantage of mentorship at the stage, I just didn't really know what I was supposed to be doing or how the process worked. I am all for being active and invested in the relationship, but I needed basic guidance to get started and not lose those precious years learning the ropes.

*Table's title, themes, and construct definitions are adapted from (19)*.

##### Launch

The start and the maturing of mentorship relationships was described as a process encompassing four main subthemes:

*Agency: Relationships Made, Not Found*. The trainee had to show initiative and boldness, to be an active agent rather than a passive recipient in the labor-intensive work of mentorship, to have a sense of time urgency by reaching out rather than waiting for things to happen, to “be proactive in that you can't expect your mentors to initiate the contact. During those attempts to connect, you need to have a pitch ready, a selling point: you are ‘selling’ (yourself) as much as you are ‘buying’ (them).” Following through with early openings was as important as showing flexibility and adaptability in shifting gears toward a productive and mutually satisfying working relationship. Pre-assigned pairings rarely proved to be as successful, no matter now well-aligned interests appeared to be. A faculty member's excitement about their work and research proved conducive to many successful relationships, and serendipity (rather than sheer luck) in being at the right place at the right time was never taken for granted by those who benefited from it. Trainees with pre-existing connections and ongoing projects by the time of their arrival to the program had a clear advantage in securing primary mentors, but relationships cemented later on could prove as productive as those set right out of the gate.

*Specificity: Where the Rubber Meets the Road*. Mentorship cannot take place in a vacuum but rather comes alive in the sharing of particular projects. Content areas of mutual interest could be obvious at times but more often took sinuous paths so as to build on, rather than replicate, a mentor's area of expertise. Trainees often felt a sense of pressure to identify a primary mentor, but many were relieved to instead end up with a network of them: “Monogamy is good in marriage, but non-exclusivity may be the better way to go with mentorship.” Indeed, finding mentors across a range of areas proved to be the norm rather than the exception. Some had “in the weeds, hands-on, directive” expertise in particular topics or methods, while others were better at “big picture” development, and yet others became specialists or problem solvers who could be mustered more selectively. Mentors who were able to zoom in and out from big picture to minute details were rare, but trainees had to make such zooming work, which often led to refining their supportive network.

*Engagement: Meaningful Connection*. Interpersonal dynamics, personality styles, and overall goodness-of-fit were essential to effective working relationships. Conducive to that end was supplanting any intimidating sense of hierarchy or distance with one of collegial partnership and being able to see and value each other as “whole persons rather than as research splices.” Demographic congruence across gender, race, or professional affiliation was valuable, when it could be found. And yet, women, underrepresented minorities, and physicians working in PhD-predominant settings clearly had more to overcome:

Where are the women physician-scientists who are going to help me figure out how to keep my research program going? There's actually not a ton of them here.

*Generativity: Setting High, Yet Realistic Expectations—and Shifting to Sponsorship*. Effective mentorship was viewed as a two-way street, in which both partners could contribute to each other's growth. Indeed, through the interconnection of like-minded mentees, the process became a multiple-way street, in which the support and guidance of peers led to a virtuous cycle of personal and scientific enrichment:

We review each other's grants. We go through difficult cases, we gripe about things. All of us are at a similar stage in our development. I think the peer group sometimes is the best place to go to, just because comradery is so very important.

Mentors were in a unique position as social “matchmakers,” making introductions within the home institution and well beyond. Effective mentors were able to enlarge the reach and specificity of the trainee's network. At their best, mentors embodied generosity by becoming not just rainmakers but sponsors. They did so not only by finding opportunities for funding, presenting, writing, or applying for a position, but also by encouraging their charges to take advantage at such developmentally enhancing junctures. Such mentors helped navigate what could otherwise become an overwhelming number of opportunities; they praised and critiqued progress; they guided and pressed gently; but they never pushed to the breaking point:

I think the folks who end up sticking it through with research often are those who have had generous mentors.

##### Orbit

We identified three discrete subthemes related to “attaining orbit,” the ability to gain professional and scientific independence and to move on after postgraduate training:

*Harnessing Potential: Carving Our Own Paths*. A mentor's ability to see the potential of their mentees and help unleash it was described as “game-changing, if not life-changing.” Trainees and graduates uniformly concurred on the critical role the program and its mentorship components had in shaping their professional trajectories, whether as subtle molding or as outright re-routing. They commented on the sheer variety of opportunities available to them, “a diversity of outlooks that so broadened my own.” They appreciated being guided to novel opportunities, as much as being advised which ones to pass over, as “Saying ‘no’ is a hard-learned developmental task.” As they found their way, many commented on their first independent forays or the intoxicating excitement and positive feedback of early successes:

I would have never thought that I was ready to write a paper in my first few months of the program. But my mentors thought I was, and they pushed me to do it, for which I am so grateful. I've kept going ever since.

*Untethering: Role Transitions*. As they approached completion of their 6-year program, graduates thought back to their first job talk and their first job. They identified a vacuum regarding how best to think about and approach either of those new and anxiety-laden developmental tasks. Finding no suitable examples relevant to their very unique set of circumstances, they chose to fill the blanks instead: “I prepared a talk on how to get and negotiate a job, and on what a job talk should and shouldn't be. I realized there was no model or manual out there, so I went ahead and created it, and hopefully others can tweak it in the coming years.”

*Customizing: Against Equifinality*. The measurable outcomes of a program designed to produce clinician-scientists are objective: grant funding, scholarly output, scientific independence, academic positions, and national or international recognition. The ability of some participants to “catch fire” during their training or early post-training years was certainly exciting and cause for celebration. However, effective mentorship and a program true to its core values need to remain aligned to the trainee rather than to the trainee's accomplishments: there can be no universal yardsticks for “success,” nor for “independence” (a concept quite distinct from “*scientific* independence”).

##### Landing

Not all successful partnerships end at the grave, last for life, or are necessarily lengthy. A careful landing may be the appropriate endpoint for an effective launch:

*Transition, Not Termination*. When the process runs its course well, as it usually does, mentorship relationships are not eliminated like so much waste, but rather metabolized differently, as befits the nurturance that they provide. The process can then shift to a better developmental fit, perhaps as mutual consultants, advisors, or mere enthusiastic supporters. Formal terminations may be more fitting for psychotherapy than for academic mentorship, although gentle boundary-setting may be appropriate at times:

“I really love the work you're doing, but I don't think that I'm able to do this to the level that would satisfy either one of us.” He was fine with that. We still say hi when we see each other, but it's a little awkward, like breakup-awkward.

In such a breakup, as in any worthwhile human relationship, internalization is the proof of its success: mentorship often comes to an end, but the meaningful relationship it is based on can long endure. In fact, the best way to close the cycle of mentorship is to pay it forward by transmitting the internalized lessons of the mentor to a new generation of learners.

##### Woes

There are many ways in which mentorship experiences can go awry, which we distilled down to two subthemes. Although such instances were less common in our sample than were the successful relationships, they represented an outsized burden that for some led to a premature departure from research altogether. Even those participants who navigated mentorship successfully would have appreciated the opportunity to have candid discussions on “how to make difficult decisions, like leaving a challenging situation, without having to worry about career consequences.”

*Vulnerability: Neglect, Attrition, Competition*. No matter how collegial the relationship, mentorship in an academic setting takes place within a hierarchical setting of career advancement. A mentor has real power over the mentee's progression, no matter how gently such power is wielded. Participants spoke of the position they could find themselves in

…when you open yourself up to someone and devote everything to something: that's when you're most vulnerable and you can get hurt the most.

The range of suboptimal mentorship experiences included examples of *neglect* (“not engaged or responsive,” “had to track them down,” “exclusively deadline-driven in responses”) and *attrition* (“too busy to keep it going,” “removed from and uninterested in my day-to-day realities,” “platitudes and bland encouragement were not helpful”). Some connections felt too transient to lead to meaningful connection, others left trainees feeling unwelcome, and yet others were hurtful through unsupportive criticism over not having a sufficiently specific project.

*Competition* took two different forms: in one, there was a sense of *working for*, rather than *working with*, a mentor, a situation in which their respective goals could become conflated, usually to the mentee's detriment:

My mentor was too busy to spend time mentoring me. It was an approach that came across as a curt “come in and get to work.” Only later, once I was close to the finish line, did he pay close attention.

More often, and on several instances, the competition was external, as when different institutions recruited mentors away, leading to destabilization or frank disruption to ongoing projects.

*Ejection: When All Else Fails*. No experiences of outright abuse were reported during our interviews, although they can certainly happen in the mentorship domain. This may be our good fortune or be due to the early recognition of mounting troubles. For example, trainees described acting on their apprehension by ending mentoring relationships gone wrong (“I had an uncomfortable weight every time we met,” “I left feeling needy, almost groveling,” “I ultimately realized it was an unproductive use of my time”). Formalizing an end to such challenging relationships proved helpful, and invariably required involvement and support from program leadership:

Maybe I could have been more proactive and assertive, or switched sooner out of a bad situation and not spun my wheels, realizing things were not going anywhere. In the end, I am glad to have cut my losses and moved on. I certainly appreciated the support, as the last thing I wanted was any fallout.

#### IV. Charting Course

The last domain encompasses four themes: (1) Focus, (2) Method, (3) Calling, and (4) Creation, as outlined in Table 5 and described below.

**Table 5:** Charting a professional course.

**Theme**	**Questions for a clinician-scientist's self-reflection**	**Representative quote(s)**
Focus	*What* do I want to know?	I have always had a pretty clear vision of what I wanted to do. I haven't deviated too much over the past 20 years, but science has, which is exciting.
Method	*How* do I want to know?	I've zoomed out and thought about the skills I wanted to learn, rather than zero in on any entrenched content.
Calling	*Where* can I best serve?	I become uncomfortable when I have to talk about the work because I don't want the work to become about me. It's a distinction that's personally important because in our work, there is a risk of ourselves taking primacy or over the patients we're supposed to be serving.
Creation	*Which* unique position will I hold someday that does not yet exist?	And now I'm in this highly technological space, where I'm doing work with engineers and developing biosensors and working on app development and things that a traditional child psychiatrist does not and would not do. And because I'm a researcher I've had the space to pivot to that new space while still working within clinical child psychiatry populations.

*Table's title and themes are adapted from (20)*.

##### Focus and Method

Reflecting back on his prodigal career, the late Donald J. Cohen articulated how he had very early on zeroed in on a specific disorder (his *focus*, his *what*) and on a specific toolkit (his *method*, his *how*):

I had a different goal—to pursue a narrow phenomenon to its roots. What if we could learn everything about a simple tic—where did it come from, what made someone vulnerable, what neurons and neurochemicals were involved, how did it get expressed or held back? How did other children feel about a child with tics, and how did their feelings make a child feel about himself? I had outlined a program of research (21).

The hallmark of the *focus* question in charting a course is being able to settle on a given diagnosis (Cohen had two main ones, autism and Tourette's syndrome). The content and aim of a career are its *what*: a commitment to a specific phenomenon (or condition, disorder or population; or, beyond the specificity of psychiatry, to any circumscribed entity or topic).

By contrast, the *method* question pertains to discrete approaches at the core of someone's expertise (Cohen had several of them: developmental psychopathology, psychoanalysis, and neurochemistry, among them). The means to pursue a question are its *how*, a powerful approach in that it can be applied to multiple goals at once.

The challenge for many clinician-scientists early in their trajectories is in liking too many *whats* but not any one of them so much as to want to make it their own. The humble tic, by virtue of being small and narrow, had proven irresistible for Cohen, a perfect soil on which he went on to plant and harvest through the methodological *hows* of biology, genes, imaging, and psychoanalysis. No such luck for many, perhaps most: “if I settle in on this choice, will I close myself off prematurely to other opportunities?” “just how narrow do I have to become in my interests to make my research career happen?” “am I exploring shifting interests or just dabbling aimlessly?”

The focus and method constructs may be best fitting for those pursuing careers in “uppercase-R Research,” aiming to be principal investigators, leaders of programs that create new knowledge; in brief, all-in scientists or clinician-scientists. Having a well-defined focus-method pairing may in fact be required for success in Research, particularly when assessed through clearly delineated metrics that are either fiscal (NIH priority scores, dollar amounts in grant funding) or academic (scholarly output, bibliometric indices).

However, trainees need to recognize that finding an optimal focus-method pairing can take time, involve fits and starts, dead ends, cul-de-sacs, and shifting of gears. The process can be as rational and deliberative as it can be sculpted by chance encounters and serendipity. Aspiring clinician-scientists need to be not just patient, but self-compassionate as well with what can be a lengthy process rife with normative, rather than exceptional, disappointments and rejections. Moreover, clinical research in child psychiatry has added complexities to consider, particularly around the “harder” and “softer” sides of its knowledge base:

…there's so much diagnostic fuzziness. And then in spite of that, many psychiatric disorders are so highly heritable. that I think it's really exciting, tying something so precise and quantitative to something as fuzzy and ill-defined.

In turn, program leaders need to be cognizant that all trainees, no matter how seemingly settled on a given direction, will need support and guidance in order to balance the breadth of their educational requirements with the depth necessary for research. Additionally, a proportion of candidate may not settle ultimately on a clearly defined and explicit research goal; some may be fulfilled with “little-r” research projects (as in education, training, or quality improvement, for example). It behooves program leaders to help each of their trainees find a path that is fitting and satisfying, a path that is responsive to their true calling.

##### Calling

By virtue of their long training, aspiring clinician-scientists have been socialized to set out goals and pursue them doggedly. They would not be physicians today, let alone pursuing such advanced education, had they not set out to master 5- and 10-year plans and long to-do lists. Given this professional upbringing, it is only natural that they are keen on pursuing targeted goals. Noble and worthwhile as this approach is, we would argue that we have done much less for our trainees when it comes to heeding the calls coming their way. Overtly centered on *what* and *how* to pursue, we all too often collude in losing sight of the *where* and the *who* reaching out to them. To misquote JFK, they should not ask what they can do for their country, but rather listen to what their country may be asking of them.

The term “calling” is usually understood in the sense of altruism, of doing something for its own good rather than for the rewards it may confer. In the case of medicine, for example, calling may be less about wanting to fix (as this would entail that our patients are somehow broken), to cure (as we often manage instead, particularly in the case of chronic conditions), or to benefit (financially or otherwise). Instead, calling should be construed around a wish to *serve* others: to ease their suffering when possible, and to bear witness and accompany them along the way even when not.

There is another way of conceptualizing “calling” —a more literal way, one more akin to listening. Each one of our trainees has to identify where it is that they are geographically, institutionally, and professionally situated at any given time: weighty decisions regarding the *where* and the with *whom* to build a residency, a fellowship, or a longer-term settled life. These places and individuals can, and often do, change over time. It is perfectly acceptable, normative in fact, to change “teams” or affiliations. But once settled in a given setting, the key is to listen attentively to what it is that may be missing, to what that location, those colleagues, and that community may be lacking. With the serious workforce shortage in child and adolescent mental health across our country and the world, clinician-scientists can be certain that there will be much that is needed and will be asked of them. It is up to them to listen in, as multiple fronts are sure to be *calling* on them.

As child and adolescent psychiatry graduates, the search for those elusive settings may be actually rather simple, the answers laying hidden in plain sight all along. Their focus is on children, adolescents, their families and the communities in which they live; their method, the vast toolkit of the profession. The two axes of their focus and method can thus effectively ground and place them. As they settle on a given setting, they can set out to find out what in its lineup may be lacking. Each and every setting will need and call upon them; it is up to them to heed the call—and for program leaders to help them heed it. Whether at a clinic, school, hospital, research lab, or any other setting; and at whatever level of training or years after graduation, everyone participating or leading the program had better listen up, for the call is surely out there.

The source of the call may have been very personal for some, who became invested through personal or familial psychopathology: “it is more than defensive sublimation: trying to address what ails us or our loved ones is a noble and worthwhile effort.” Others may have gravitated more generally to the suffering of fellow humans:

…none of these things, fortunately, were in my family. No one had a hard time like this, so it wasn't personal for anyone close to me. It was really being around the kids in the clinic through my work in medical school and residency that got me interested. This is a disorder that really needs help. Children and their families need help. We need better treatments.

The social and racial unrest that became more overt in recent years, and particularly following the 2020 syndemic of COVID-19 and racial inequity, gave a renewed urgency to “community relevance, not just publications when our house is on fire.” The opportunity—indeed, the responsibility—to address communal and societal challenges, “to telescope out from the single child,” “to communicate with the public and share hope,” “to take on the messy realities out there” was invigorating for yet others:

I only knew about community-based research because I got a degree in public health, where everybody knows about it as common knowledge. But as a psychiatrist, as a physician, it is not yet part of our standard vocabulary. I want to address that gap.

##### Creation

Identifying a job and devoting one's life to it is a formerly effective approach to employment that has diminishing relevance in today's world. And what is true for workers of factory plants, rustbelts, or mechanized farms is similarly applicable to clinician-scientists, who are overwhelmingly likely *not* to remain in a single academic setting for life. They should celebrate this fact. They should embrace vast new opportunities. And they should be instrumental in their creation.

*The job that you will have someday does not yet exist*. When we first heard these words from well-meaning mentors, we were scared silent. Inventing a future job from scratch appeared too ill-defined, amorphous, and inchoate a proposition for some as comfortable with structure, security, and predictability as we were. However, time proved the prescient wisdom of those words, as we each have seen ourselves and so many of our colleagues reinvent themselves professionally, moving in a way that hardly seemed linear at the time, but that in retrospect appeared so natural—predestined almost.

These words can prove frightening to young trainees, just as they once scared us; alternatively, they may come across as too abstract to be of much use. To that end, we have come to suggest a more direct and even prescriptive approach, a thought experiment for trainees to entertain and revisit periodically:

Their very own creation, the one tailored to their unique needs and talents, this one-of-a-kind bespoke, may not yet exist. It is up to them to design this beautiful “garment” of theirs. And if they can think of no way of doing so, we invite them to a challenge that helped us as first-time tailors. For starters, think of how to give back. Whether their reach is local or global; whether it centers on patients, parents, or professional colleagues; whether it is through research, volunteer, educational, or any other means, they *can* give back. And when they think back to the first three domains they worked so hard to claim as their own, remind them they have valuable and rare skills: they have a focus (children), a method (their child psychiatry toolkit), and a team to call their own (the setting or location calling on them). The one thing remaining is to have those hard-earned skills meet with a commitment to service: it is at the intersection of skills and service that they will be able to give back, and in so doing, to live a more fulfilling life, both professionally and personally:

…the program allowed me to see myself as a leader and also see that maybe the problem of why I hadn't seen myself as a leader or as leading a lab before was because I thought there were probably enough of those people around. The program helped me realize we were the ones we had been waiting for.

## Discussion

The findings from this qualitative study of an integrated clinical and research training program, including the four-domain model we developed, can have practical utility for stakeholders involved in the education of clinician-scientists, particularly—though not exclusively—those in the field of child and adolescent mental health. To illustrate the model's practical utility, we provide examples of lessons learned in going through this exercise. Finally, we address the study's limitations and future opportunities.

### Practical Utility of the Four-Domain Model

A useful way of approaching the model is through putative questions that relevant stakeholders could ask themselves by using the domains as points of departure:

#### Individual

A medical student contemplating the momentous decision of which program to apply to may ask herself: “Is becoming a clinician-scientist right for me?” The themes and subthemes in the first domain may be a useful rubric through which to self-reflect and have additional and specific criteria to better answer the question. For example, “How comfortable will I be in developing an independent project of my own?” “Do I want to *explore* my research interest, or am I ready to *commit* to it?” In turn, program directors looking to identify the most suitable and best-fitting applicants may consider, “What traits are we looking for in our clinician-scientist trainees?”, “What can we learn about them above and beyond traditional metrics?” Questions such as these can be incorporated as a complement to domains of Career Construction Theory (CCT) (22), as was recently applied to interns who chose careers in psychiatry (23): *concern, control, curiosity, confidence*, and, of particular relevance to future investigators, *contribution*.

#### Program

Once set on a clinician-scientist direction, a candidate can go on to ask: “Is *this* the right program for me?” “Does its balance between structured and unstructured components work for me?” “Just what is the right amount of structure and freedom for me to thrive in?” For their part, directors and developers can contemplate, “How can we improve and optimize the program?” In the next section, and based on our own experience, we provide two concrete answers to this question.

#### Mentorship

By the time a trainee is settled on a program, en route to it in fact, the questions should swivel to how to maximize the training experience: “Who in the program can help me make the most of my time there?” “What is the right number and constellation of mentors for me?” It could be limiting or even risky to rely on a single mentor, yet an overabundance of mentors could become dilute, confusing, or simply too time-consuming. The sequence and timing of mentor outreach is important, as is bearing in mind that even brief mentorship encounters can be incredibly productive. For their part, mentors and would-be mentors can consider, “How can I be the best mentor for this particular trainee?” or “How can I optimize my experience and background toward the benefit of someone else?”

#### Charting Course

The fourth domain does not lend itself to as clear a distinction among the perspectives of trainees, mentors, or program directors. And that is because the charting of a career's course should be a lifelong quest for each and every one of us. In answering the key question “Who do I want to become?” words adapted from Frederick Buechner (24) can prove essential: *Where your deep gladness meets the world's deep hunger is where you should be: there lies your vocation*. The reply we would give our charges works just as well when self-directed: Don't let them worry alone if they can't figure it all out at once; they may get there sequentially, but then again, they may not. Help them anticipate and adapt to change and the certainty of vicissitudes. Support them during the time it will take to find out what gives them real joy, what is the deep gladness looking to break out of them. Help them embrace, find, or reconnect with their true vocation. They will find it at the crossroads of their deep gladness and the deep hunger out there—a hunger so keenly felt by those they are privileged to serve.

### Applying the Model: Two Examples of Program Enhancement Opportunities

Participants in the study showed remarkable candor. It was clear that by not holding back in either enthusiasm or critiques, they were providing constructive feedback to a program they care deeply about, whether as trainees or alumni. However, there were two aspects in which they seemed more circumspect, finding it harder to share with quite the same level of comfort or openness: their personal mental health, and doubts about long-term commitment to research. Rather than pressing the issues during the interviews, we have taken them as a call for action.

#### Healer, Heal Thyself: Addressing Physicians' Mental Health and Well-Being

Several participants shared their involvement in psychotherapy, commenting on how useful it had been to their clinical work or personal development. Notably, the experience was described using terms more relevant to *training* than to *treatment*, e.g., “growth-enhancing,” “enriching,” “useful,” “being on the other side.” Moreover, no one overtly acknowledged personal psychopathology, which would represent a major statistical anomaly, considering the high prevalence of psychiatric illnesses in young adulthood. Indeed, physicians have rates of depression even higher than the lay public (25). As a group, physicians are notoriously poor at identifying or addressing their own mental health needs, and psychiatrists may fare no better than other specialists (26). If anything, the experience in our study is paradigmatic of a much larger crisis affecting the house of medicine.

Outlining a comprehensive approach to addressing the mental health needs of our program's trainees is beyond the scope of this article. Still, general guidelines that we have started to institute are pertinent. First, it is critical to not conflate exhaustion or burn-out with mental illness; discussions about the former are certainly important and can lead to recognizing the latter. However, all too often, depression and other serious conditions can go unrecognized and untreated, construed instead as externalities with bland solutions like wellness initiatives. Second, we will continue to provide release time for therapy, not only as the training enhancer that it certainly can be, but embraced without shame or stigma as treatment. Third, our institution's office of graduate medical education has streamlined the confidential referral to appropriate treatment. Finally, it behooves training leaders and senior faculty to lead by example in sharing their own lived experiences with mental illness:

The medical adage to “see one, do one, teach one” also rings true for judgment and self-care around mental health. However, most [trainees] do *not* see their attendings talk as casually about how an antidepressant or psychotherapy helped them get over depression as when a steroid injection or physical therapy helped their tennis elbow. In turn, they then do *not* comfortably admit feeling depressed, and in turn will *not* teach the next generation of trainees to get support when needed. As a result, stigmatized views about mental illness become entrenched and internalized, and each new class of trainees is flooded with lots of *talk* about self-care, without the commensurate role-modeling *walk* by their superiors (27).

In earlier work we have empirically demonstrated the salutary effects of sharing lived experiences with medical students (27, 28) and with physician assistants (29). We don't necessarily need to conduct a similar study with clinician-scientist trainees in CAP, but it would be inexcusable not to apply the lessons we have learned: sharing histories of personal vulnerability by senior faculty can lessen stigmatized and self-stigmatized views of mental health and normalize help-seeking behaviors among trainees.

#### Opting in Is Not Copping Out: Guidance and Support in Selecting the Right Road

A number of participants alluded to “buyer's remorse” early in the program, or to recurrent, gnawing doubts about their decision to become clinician-scientists later on. Such questioning was not universal but certainly not rare either. Further, the discomfort with even skirting around the edges of the issue was evident. Trainees with as stellar backgrounds, trajectories, and promise could be paradoxically set up for the shortcomings of a medical culture and implicit hidden curriculum that make trainees feel like there is no room for mistakes in medicine, space for flaws in their personal makeup, or for rerouting their professional trajectories. Being in a high-pressure, outcome-driven, and demanding environment can set the stage for imposter syndrome feelings, narcissistic injury, or fear of being “outed” as somehow lacking.

As leaders, it is incumbent on us to prioritize our trainees' developmental needs and well-being over our program's outcomes: the *person* must always take precedence over their *product*. Concretely, we need to make it clear that, for certain trainees, selecting a different path should not be considered a failure when either the push away from science, or the pull toward something else is too strong to be ignored:

As I considered leaving research after so many years of training, I struggled with the idea of letting people down…I felt that transitioning to a clinical career could cause irreparable damage to relationships with the very people that supported and believed in me, leaving me isolated. Leaving would mean foregoing rich academic resources to conduct research that could—potentially, elusively—lead to treatments with an impact on the lives of so many. Instead I opted to dedicate myself to clinical work that would touch a significantly smaller number of people but would do so at a timescale and community immediacy I longed for. At the end of the day, it was those very mentors, family, and peers who encouraged me to apply the knowledge I had gained in the way that was most fulfilling for me…I did not “leave” anything. Instead, I arrived at a way of leveraging my personal story, my training, and my love for improving the lives of youths and families in my small, under-resourced community. I am at peace (30).

## Limitations

First, we recognize that, as direct stakeholders directly involved in the AJSP, our views may be biased in a way than an outsider's might not be. To that end, we do not consider this study a formal evaluation of the program, for that purpose relying instead on the annual metrics set out by appropriate governing bodies, and on reporting requirements from funding agencies. Moreover, we have an external advisory board, and additionally conducted a consultation with outside experts after the initial decade of the program. We should also note that there was a substantial amount of feedback about programmatic granularities that we left out of this analysis and which may still prove useful in our efforts to improve the overall quality of the program in moving forward. Second, we did not interview stakeholders other than current trainees and graduates from the AJSP; the perspectives of program leaders, mentors, and external advisors are thus lacking. Finally, we recognize the limitations inherent to a single-site study conducted in a developed country and high-resourced setting. However, we consider having done well on the trustworthiness of our report as reflected by two qualitative constructs (31, 32): (1) *credibility*, or the plausibility of our descriptions being recognized by its participants (a concept akin to internal validity); and (2) *transferability*, or the applicability of findings to other settings and programs (akin to generalizability or external validity).

## Conclusions

In summary, we conducted a qualitative study based on individual interviews of all participants enrolled over the course of the 17-year history of a program designed to develop clinician-scientists dedicated to child and adolescent mental health. We developed, through iterative thematic analysis, a four-domain model resulting from the intersection of a developmental perspective (spanning professional or personal spheres) and a reflective direction (outward- or inward-facing vantage points). The model can be fruitfully applied to individual or programmatic evaluations and can help identify, refine, and replicate programs that are urgently needed to increase the working force of clinician-scientists dedicated to advancing the child and adolescent mental health agenda.

## Data Availability Statement

The original contributions presented in the study are included in the article/supplementary material. Further inquiries can be directed to the corresponding author/s.

## Ethics Statement

This study involved human participants and was reviewed and approved by the Yale Human Investigations Committee (Protocol # 2000027895). Written informed consent for participation was not required for this study in accordance with national legislation and institutional requirements.

## Author Contributions

AC and AM designed the study. AC conducted the interviews. AC and OH took the lead in analyzing the transcripts. AM drafted the first version of the manuscript and is responsible for the integrity of the data and the analyses. All authors reviewed and contributed to working drafts and approved the final submitted version.

## Conflict of Interest

The authors declare that the research was conducted in the absence of any commercial or financial relationships that could be construed as a potential conflict of interest.
